# Symptomatic infantile helicobacter pylori gastritis infection in indigenous african infants: a case series

**DOI:** 10.11604/pamj.2014.19.83.4801

**Published:** 2014-09-25

**Authors:** Oliver Ombeva Malande

**Affiliations:** 1 Department of Paediatrics and Child Health, Egerton University, Kenya

**Keywords:** Infantile, helicobacter pylori, gastritis, infection

## Abstract

*Helicobacter pylori* gastritis infection rate increases with age. Higher rates have however been reported among young people in the developing countries of the world. The infection however has rarely been reported in infants, especially in Africa. This case series describes three cases of *Helicobacter pylori* gastritis infection as diagnosed in three infants. The goal is to raise the suspicion index of medical practitioners about the possibility of this this infection among infants who present with suggestive symptoms. On three separate occasions in 2012 and 2013, three ill, indigenous, black African female infants aged 4, 6 and 7 months, were brought to hospital with symptoms ranging from fever, refusal to feed, diarrhoea, restlessness, vomiting and irritability. In each case, systemic examination findings were unremarkable. After several laboratory investigations, each infant was found to have *Helicobacter pylori* infection following positive blood antibody (using Tell Me Fast H. Pylori antibody serum and Plasma test manufactured by Biocan Diagnostics Canada) and fecal HpSA ImmunoCardSTAT antigen tests. Repeat stool antigen test was negative in each case after completion of the recommended triple therapy. *Helicobacter pylori* infection has been rarely reported among infants. This case series highlights the need for health care providers to have a high index of suspicion so that infants with suggestive symptoms, especially in settings with high *Helicobacter pylori* colonization prevalence can be evaluated for *Helicobacter pylori* gastritis infection.

## Introduction

*Helicobacter pylori* is a bacteria that causes gastritis in humans [1]. Though the rate of infection increases with age, higher rates have been reported among young people in the developing countries of the world, where poor sanitation is thought to contribute to an overall high prevalence of *Helicobacter pylori* infection [1–3]. The infection however has rarely been reported in infants, especially in Africa. In this case series, we describe three cases of *Helicobacter pylori* gastritis infection as diagnosed and treated in indigenous female black African infants from Uganda. We intend to highlight the need for attending medical practitioners to think about and indeed look out for possibility of *Helicobacter pylori* infection in infants. These infants often, present with symptomatology that may suggest other different childhood infections. Our concern mainly involves, developing low income settings where *Helicobacter pylori* colonization has previously been described generally among children, but rarely in infancy. Affected patients from these poor settings would therefore would therefore benefit from; a more focused and targeted approach to diagnosis that quickens the treatment of this disorder while reducing the overall costs of investigations and treatment for a patient population that often cannot afford the usual high costs of health care.

## Methods

The first case was a four month old indigenous black African female infant, with a two week history of low grade fever, two days of diarrhea, vomiting and refusal to breastfeed. There was no cough, difficulty in breathing, painful micturition, convulsions or abdominal distension. The mother had introduced the child to complementary feeding with both cow milk and infant formula three weeks to the onset of symptoms. The child was restless, irritable, dehydrated, without fever and unremarkable systemic examination findings. The general nutritional status was good. Investigations revealed normal Complete Blood Count findings, negative slide for malaria parasites; non-reactive Widal and Brucella antibody tests and a POSITIVE *Helicobacter pylori* blood antibody (using Tell Me Fast H. Pylori antibody Test for serum and Plasma test manufactured by Biocan Diagnostics Canada) and stool antigen HpSA ImmunoCardSTAT tests.

The second case was a six month old indigenous black African female infant, who presented with one month history of recurrent abdominal pain, diarrhea, restlessness and crying a lot. There was no history of cough, fever, painful micturition, convulsions, vomiting or abdominal distension. She was afebrile, not dehydrated, with unremarkable systemic examination findings. Investigations revealed normal Complete Blood Count findings, negative slide for malaria parasites, non-reactive Widal and Brucella antibody tests, and a POSITIVE *Helicobacter pylori* blood antibody (using Tell Me Fast H. Pylori antibody Test for serum and Plasma test manufactured by Biocan Diagnostics Canada) and stool antigen HpSA ImmunoCardSTAT tests.

The third patient was a seven month old indigenous black African female infant, who presented with a three days of fever, watery diarrhea and postprandial vomiting, with no history of cough, abdominal distention, convulsions or painful micturition. She was febrile and dehydrated, with normal Complete Blood Count findings, negative Widal and Brucella tests, and a negative Blood slide for malaria parasites. Further tests revealed a POSITIVE *Helicobacter pylori* blood antibody (using Tell Me Fast H. Pylori antibody Test for serum and Plasma test manufactured by Biocan Diagnostics Canada) and fecal antigen HpSA ImmunoCardSTAT tests. Written informed consent was obtained from the parents of the infants described in this manuscript for publication of this case series. Copies of the written consent forms are available for review by the Editor-in-Chief of this journal.

## Results

Each of these three indigenous Ugandan infants improved on a combination of IV/oral omeprazole, oral clarithromycin and IV/oral metronidazole, with complete resolution of symptoms after completing a fourteen day course. Dehydration was managed as per World Health Organization guidelines [5]. Repeat *Helicobacter pylori* stool antigen test after treatment was negative in each of the cases.

## Discussion

The prevalence of *Helicobacter pylori* as a common cause of infection has been previously described [1–4], with numerous studies showing a high colonization prevalence among people in low-income countries and colonization early in life [2–4] indeed, a community-based cross sectional survey in apparently healthy children aged 0-12 years in urban Kampala, Uganda [5]; found a high colonization prevalence of 44.3%, with a common infant population early colonization of 28.7%. This survey only considered healthy children; with no symptoms of disease. While findings in this survey point to presence of early *Helicobacter pylori*colonization, early symptomatology and active disease has rarely been described, especially during infancy - an age group that can be easily overlooked.

The finding of active *Helicobacter pylori* related infection in the cases reported in this series point to a greater need to respond to the recommendations of the Kampala survey [5] that the impact of *Helicobacter pylori* colonization on children′s health in Uganda needs to be further clarified. The response to treatment specifically targeting *Helicobacter pylori* disease in the cases reported here strengthen the correctness of the diagnosis of *Helicobacter pylori* gastritis infection, though with limitations of failure to rule out urinary tract infection as a cause for the fever at presentation in case 1 and 3, (due to difficulties involved in collecting reliable urine samples), and failure to perform upper Gastrointestinal (GI) endoscopy to identify any gastritis related ulcerative lesions. It is difficult to perform upper GI endoscopy in infants; and we do not have the equipment or the trained personnel to perform this investigation in the setting where these infants were treated. However, the diagnosis of these cases is correct, especially considering that we used confirmatory monoclonal antigen fecal HpSA ImmunoCardSTAT test, which has a high sensitivity, specificity, accuracy and reproducibility [1, 3, 5–7], and is easily affordable for the poor and low income settings especially in Africa. Studies show that most children colonized with *Helicobacter pylori* are asymptomatic [3–5], and this could contribute to a low index of suspicion by attending health care providers. Most of these studies also focus on general paediatric population with little emphasis on infants.

Presentation of *Helicobacter pylori* gastritis in infants is nonspecific at best, with abdominal pain in children being a non-specific symptom [8]. While studies show improvement in abdominal pain in children with *Helicobacter pylori* gastritis after triple therapy, a recent double-blind controlled trial did not confirm this finding [8], while a meta-analysis found conflicting evidence for association between *Helicobacter pylori* and epigastric pain [9]. Whereas a positional statement of the North American Society of Pediatric Gastroenterology, Hepatology, and Nutrition did not find convincing data supporting routine testing for *Helicobacter pylori* in children with abdominal pain [3], these three cases of *Helicobacter pylori* infection in infants show that attending health care providers should suspect and test for possible *Helicobacter pylori* infection in symptomatic cases, and more so in infants where presentation is non-specific and abdominal pain (recurrent) may not be a clearly identifiable symptom. Based on recommendations [10], each of these three cases responded successfully to triple therapy treatment, and had subsequent negative repeat stool antigen test results. In the low income and developing countries of the world, with high colonization rates for Helicobacter pylori, it would suggest that focused and affordable/low cost early diagnosis [7] (eg the tests used in diagnosis of these cases) of symptomatic *Helicobacter pylori* infection would reduce the overall costs of health care, since other additional and not so relevant investigations would be avoided, and the overall period to arrive at accurate diagnosis would be reduced.

*Helicobacter pylori* antibody levels may remain elevated for a long time; however, though each of these cases showed negative antibody test 7-10 days after completion of treatment; we relied on negative stool antigen tests with resolution of clinical symptoms to define cure. There is the possibility of co-infection with other fever causing conditions including viral infections contributing to the presence of symptoms in these infants; however, the likelihood of other etiology apart from *Helicobacter pylori*is very low, as shown by positive *Helicobacter pylori* fecal /antigen test at diagnosis and concurrent resolution of symptoms following specific treatment targeting *Helicobacter pylori* infection. It has been suggested before that *Helicobacter pylori* infection can resolve spontaneously in children, however this is more likely in situations of colonization in absence of active infection accompanied by symptoms of disease that warrant medical attention.

## Conclusion

*Helicobacter pylori* infection has been rarely reported among infants. This case series highlights the need for health care providers to have a high index of suspicion so that infants with suggestive symptoms, especially in settings with high *Helicobacter pylori*colonization prevalence be evaluated for *Helicobacter pylori* gastritis infection.
